# Tau and atrophy: domain-specific relationships with cognition

**DOI:** 10.1186/s13195-019-0518-8

**Published:** 2019-07-27

**Authors:** Leonardino A. Digma, John R. Madsen, Emilie T. Reas, Anders M. Dale, James B. Brewer, Sarah J. Banks, for the Alzheimer’s Disease Neuroimaging Initiative

**Affiliations:** 1 Department of Neurosciences, University of California, San Diego, 9500 Gilman Drive, La Jolla, CA 92093 USA; 2 Department of Radiology, University of California, San Diego, 9500 Gilman Drive, La Jolla, CA 92093 USA; 3 Center for Molecular Imaging and Genetics, University of California, 9500 Gilman Drive, San Diego, La Jolla, CA 92093 USA; 4 Department of Psychiatry, University of California, San Diego, 9500 Gilman Drive, La Jolla, CA 92093 USA

**Keywords:** Tau, Atrophy, Thickness, Positron emission tomography, Magnetic resonance imaging, Neuropsychology, Cognition, Surface-based analysis

## Abstract

**Background:**

Late-onset Alzheimer’s disease (AD) is characterized by primary memory impairment, which then progresses towards severe deficits across cognitive domains. Here, we report how performance in cognitive domains relates to patterns of tau deposition and cortical thickness.

**Methods:**

We analyzed data from 131 amyloid-β positive participants (55 cognitively normal, 46 mild cognitive impairment, 30 AD) of the Alzheimer’s Disease Neuroimaging Initiative who underwent magnetic resonance imaging (MRI), flortaucipir (FTP) positron emission tomography, and neuropsychological testing. Surface-based vertex-wise and region-of-interest analyses were conducted between FTP and cognitive test scores, and between cortical thickness and cognitive test scores.

**Results:**

FTP and thickness were differentially related to cognitive performance in several domains. FTP-cognition associations were more widespread than thickness-cognition associations. Further, FTP-cognition patterns reflected cortical systems that underlie different aspects of cognition.

**Conclusions:**

Our findings indicate that AD-related decline in domain-specific cognitive performance reflects underlying progression of tau and atrophy into associated brain circuits. They also suggest that tau-PET may have better sensitivity to this decline than MRI-derived measures of cortical thickness.

**Electronic supplementary material:**

The online version of this article (10.1186/s13195-019-0518-8) contains supplementary material, which is available to authorized users.

## Background

Alzheimer’s disease (AD) typically presents clinically as an amnestic disorder with subsequent progression to other cognitive domains such as visuospatial abilities, language, and executive function [1]. The domains of cognition rely on distinct cortical systems, where distributed and specialized brain circuitry serves associated cognitive functions [2]. Degeneration and dysfunction of these large-scale cortical systems parallel the decline of specific cognitive domains observed in dementia [3].

Pathological events in AD are considered to cause the clinical manifestations through sequential and synergistic build-up of pathological proteins, amyloid-β (Aβ) and tau, in the brain and neurodegeneration. Although there is some evidence that elevated levels of Aβ are associated with future cognitive decline [4], regional Aβ is generally a poor correlate of cross-sectional cognition [5, 6]. Tau and neurodegeneration, on the other hand, have been shown to be closely linked to deficits in cognition [7, 8]. While the associations with tau have been determined through studies of post-mortem brain tissue [9], we are now able to study them in vivo using PET ligands [10, 11].

Autopsy data indicate that the spread of tau pathology follows a progressive sequence [12] consistent with the observed clinical course in AD: tau starts in the medial temporal lobe regions that are responsible for learning and memory, then into the rest of the cortex to affect the cortical areas that serve other cognitive domains. Initial studies using tau-binding radiotracers have recapitulated the Braak staging of tau that has been described in autopsy studies [13, 14], establishing the promise of these radiotracers for assessing, in vivo, how tau pathology relates to domain-specific cognitive decline.

Assessment of neurodegeneration in vivo has been available with high-resolution magnetic resonance imaging (MRI) measures of cortical thickness to estimate structural atrophy and with fluorodeoxyglucose (FDG)-PET to evaluate regional hypometabolism. Studies using these modalities have consistently shown that AD is characterized by degeneration in temporal and parietal cortices [15–18] in a pattern similar to tau pathology. Furthermore, in vivo studies of neurodegeneration, like post-mortem studies of tau, have revealed close associations between the distribution of neurodegeneration and cognitive deficits found in patients with AD [3, 19–23].

Taken together, studies of tau and neurodegeneration suggest that the two are closely related and that both contribute to variability in cognitive performance. The relationship between neurodegeneration and cognition across different domains in vivo has been explored extensively, but the link between tau and cognition across domains in vivo remains to be fully characterized. Studies of tau-PET in early-onset atypical variants of AD, such as primary progressive aphasia or posterior cortical atrophy, have shown that tau accumulation in cortical systems that serve specific cognitive domains is associated with impairment in those respective domains [24, 25]. Interestingly, the aggregation of tau within functional cortical systems [26] and the association of tau with cognitive loss in different domains [27] has recently been demonstrated to similarly occur in late-onset, amnestic AD. In this study, our interest was to further examine how in vivo measures of tau and neurodegeneration associate with different domains of cognition in the common, amnestic AD phenotype.

To this end, we used multi-modal imaging and neuropsychological battery data available in the Alzheimer’s Disease Neuroimaging Initiative (ADNI) to investigate the relationship between cross-sectional measures of tau, cortical thickness, and different aspects of cognition. We hypothesized that tau and cortical thickness would both correlate with cognition in a domain-specific manner, with tau demonstrating stronger associations over a wider area of the domain-relevant cortex. We tested these hypotheses by performing surface-based vertex-wise analysis and region-of-interest (ROI) analysis in a group of Aβ-positive older adults with a diagnosis of cognitively normal, mild cognitive impairment, or AD.

## Methods

### Study participants

Our sample consisted of subjects from phase 2 and phase 3 of the ADNI. The ADNI was launched in 2003 as a public-private partnership, led by Principal Investigator Michael W. Weiner, MD, with the primary goal of testing whether serial imaging, biomarker, and clinical and neuropsychological assessments can be combined to measure the progression from MCI to AD. For up-to-date information, see www.adni-info.org. Inclusion and clinical diagnosis criteria for ADNI have been described previously [28, 29]. We included subjects from ADNI who received MRI, tau-PET (flortaucipir-PET, FTP-PET), and Aβ-PET (florbetapir- or florbetaben-PET) and underwent full neuropsychological testing, and had these data available in the ADNI data repository in August 2018, when our study began. Only subjects that were Aβ positive, as determined by Aβ-PET and a standard threshold [30, 31] were included for statistical analysis to focus our study on participants who were on the AD pathophysiological continuum [32]. The Aβ-PET scan that was acquired closest in time to the tau-PET (time between Aβ-PET and tau-PET: mean = 1.12 years, SD = 1.57 years) scan was used to determine Aβ positivity.

### MRI acquisition and processing

**Fig. 1:**
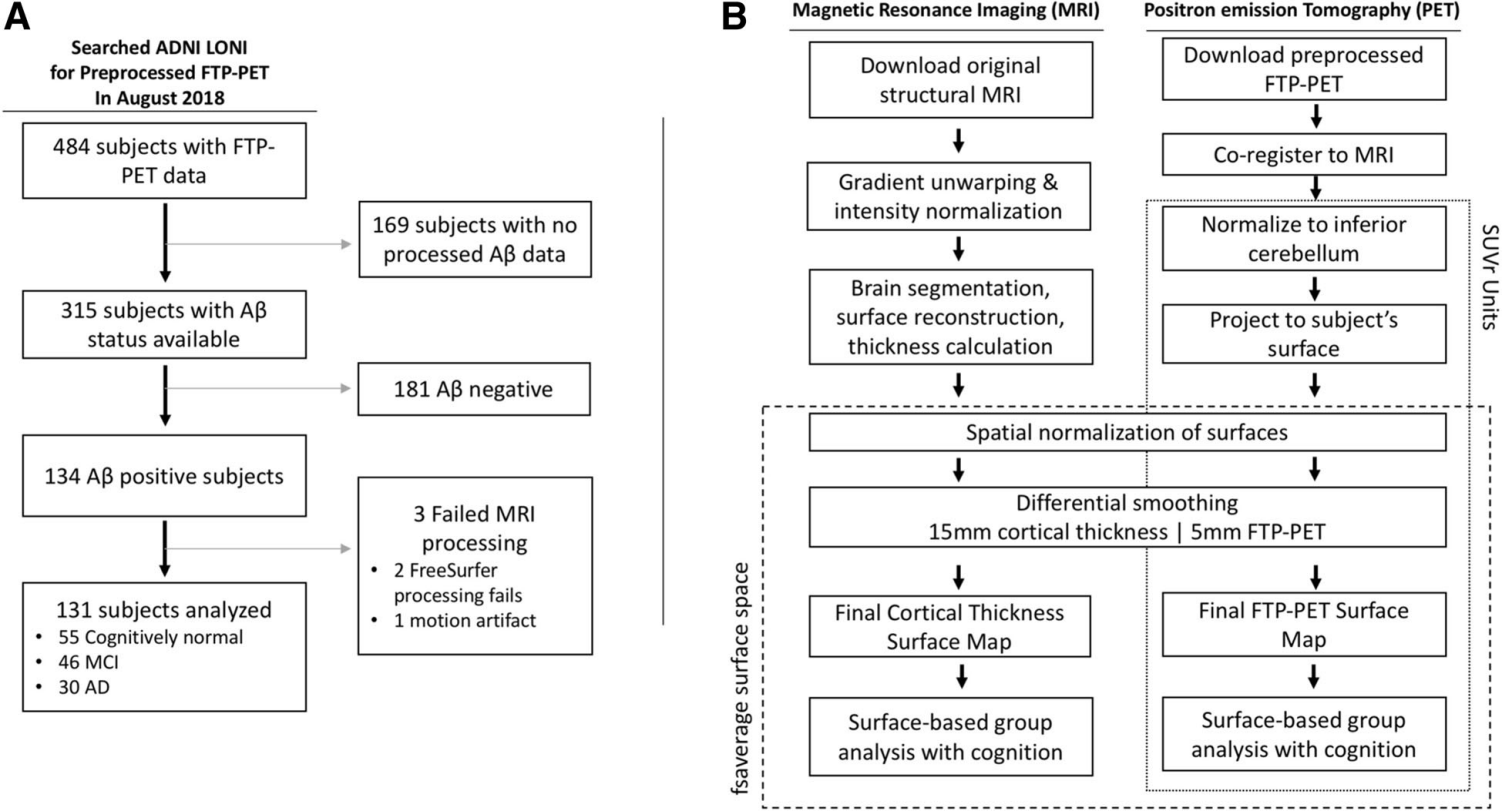
Flow diagrams for subject inclusion and processing of neuroimaging data in our study. **a** The steps used for assembling the study sample. **b** The steps used for image processing for surface-based vertex-wise analysis. Abbreviations: SUVr: standard uptake value ratio; FTP: flortaucipir; mm: millimeters

An overview of the image processing steps used in our study is outlined in Fig. 1. Briefly, raw T1 MR images were downloaded in DICOM format from the ADNI data portal. Spatial distortions in MR images resulting from gradient nonlinearities were corrected using scanner-specific parameters [33]. Corrections for nonuniformity in signal intensity were then applied [34]. FreeSurfer (version 6.0) was used to automatically segment and parcellate each structural MRI, reconstruct the cortical surface, and measure thickness at each vertex on the cortical mantle [35, 36].

We also defined each subject’s inferior cerebellar gray matter, which was used as the reference region in PET analyses (see the “PET acquisition and processing” section). The MRI was segmented into tissue probability maps with SPM12 (www.fil.ion.ucl.ac.uk/spm). Then, the spatially unbiased atlas template of the cerebellum and brainstem toolbox (SUIT, http://www.diedrichsenlab.org/imaging/suit.htm) was used to reverse normalize the SUIT template to the participants’ native space MRI.

### PET acquisition and processing

FTP-PET was conducted to estimate the extent of tau pathology. FTP-PET images were downloaded from the ADNI portal in the most preprocessed form: realigned to the first frame, averaged across frames, voxel sizes standardized, and resolution made uniform across sites [37]. These preprocessed images were coregistered to the subject’s temporally closest MRI. These co-registered FTP-PET images were then converted into standard uptake value ratio (SUVr) images by normalizing the images to mean FTP uptake in the inferior cerebellar gray matter [38]. The individual SUVr volumes were then projected onto each individual’s cortical surface model by sampling from points half-way between the white and pial surfaces.

### Processing for surface-based analyses

Prior to conducting vertex-wise analyses (see the “Statistical analysis” section), the individual thickness maps and FTP maps were brought into a common space (fsaverage space) using spherical-based registration methods [39]. Furthermore, because cortical thickness and FTP datasets differed in spatial resolution in the surface maps, differential smoothing was applied to the thickness and FTP surface maps to obtain similar spatial resolutions along the cortical mantle prior to surface-based analysis. Kernels of 15 mm and 5 mm were used for the thickness and FTP maps, respectively.

### Processing for region-of-interest analyses

In addition to surface-based vertex-wise analyses, we also performed ROI analyses using partial volume corrected (PVC) regional FTP data. The Geometric Transfer Matrix [40] approach for PVC was applied, in combination with FreeSurfer-parcellated ROIs from the Desikan-Killiany atlas [41] and SPM12 tissue segmentations as described previously [38]. For regional cortical thickness, we used the average thickness of each Desikan-Killiany ROI as outputted by FreeSurfer.

### Neuropsychological assessment

Participants underwent neuropsychological testing to assess performance across cognitive domains. We used scores from their exam that was closest in time to their FTP-PET date (time between neuropsychology visit and FTP-PET: mean = 0.60 years, SD = 0.78 years). We used various tests scores to represent the domains in question: the Rey Auditory Verbal Learning Test (RAVLT) and Logical Memory (LM) tests were used to assess learning and memory. We analyzed the RAVLT sum of all correctly learned words across trials 1 through 5 (RAVLT learning), RAVLT delayed recall, LM immediate recall, and LM delayed recall. To assess verbal fluency and naming abilities, we examined scores from the category fluency: animals and the Multilingual Naming Test (MINT). Lastly, to assess constructional abilities, processing speed, and executive function, we analyzed clock drawing score, clock copying score, and scores from parts A and B of the Trail Making Test (TMT), respectively. For all statistical analyses that involved TMT Part A or TMT Part B, scores were multiplied by − 1 so that lower scores indicated worse cognition.

### Statistical analysis

#### Surface-based analysis

We performed surface-based general linear model analyses as implemented in FreeSurfer to explore the relationships between tau and cognition, and between cortical thickness and cognition. We used surfaced-based analysis because surface-based registration offers a more accurate alignment of cerebral cortex across subjects than volume-based registration [42] and because 2D smoothing across the surface helps prevent smoothing across different tissue types in the PET data [43]. Vertex-wise analyses were performed between FTP-PET and scores on each cognitive test, and between cortical thickness and scores on each cognitive test, with regressors for age, sex, education, and time-delay between scan and cognitive test administration. Model contrasts tested the hypothesis that more tau or thinner cortex (in separate models) was associated with worse cognitive performance. A Monte Carlo simulation method [44] was implemented for cluster exclusion and multiple comparisons correction, using a cluster-forming threshold of -log(*P* value) > 4 or *P* value < 0.0001, and a cluster-wise threshold *P* value < 0.05. All statistical surface maps are displayed at a threshold of -log(*P* value) > 4 or *P* value < 0.0001 unless otherwise specified. We also performed diagnosis-stratified surface-based analyses, within CN, MCI, and AD subjects.

#### Domain-specificity analysis

Visual inspection of the FTP-cognition association maps suggested that there may exist cognitive domain-specific FTP retention patterns. To this end, we performed post hoc pairwise Meng’s tests [45] between the FTP-cognition partial correlations maps (vertex-wise *P* value < 0.005) [46]. Meng’s test compares two dependent correlations and allows us to determine whether FTP is more related to one cognitive test versus another at each point on the cortical surface. Meng’s test was performed pairwise for the statistical maps from FTP vertex analyses with RAVLT learning, RAVLT delayed recall, category fluency, and TMT part A. These 4 tests were chosen because they had qualitatively similar topographical associations with tau as for LM immediate recall, LM delayed recall, MINT, and clock drawing, respectively. To limit the number of vertices examined, Meng’s test was only performed in vertices that survived cluster correction in either of the analyses being compared.

The domain-specificity analysis was also performed for the partial correlation maps from the surface-based thickness-cognition analyses. The same subset of tests—RAVLT learning, RAVLT delayed recall, category fluency, and TMT part A—were used for the domain-specificity thickness analyses. These results are reported in Additional file 1.

#### Region-of-interest analyses

To re-capitulate our vertex-wise analysis results at the ROI level, we performed ROI correlational analyses. We computed partial correlations between regional PVC-FTP and cognitive test score, after each variable was regressed onto age, sex, education, and time-delay between the date of scan and date of cognitive test administration. This correlational analysis was done for each ROI and cognitive test pair. This analysis was repeated for regional thickness, instead of regional PVC-FTP.

To explore the question of whether regional tau was associated with cognition, independently of regional thickness, we performed the following ROI analysis. We again computed partial correlations between regional PVC-FTP and cognitive test score. However, in this case, we regressed each variable onto regional thickness, in addition to age, sex, education, and time-delay between scan and cognitive test. This allowed the examination of associations between regional PVC-FTP and cognition, independent of regional thickness. Results from our ROI analyses are displayed in correlation matrices in Fig. 5.

In these analyses, we excluded FreeSurfer-defined regions that correspond to Braak stage 6 [13] as they likely exhibit little variability in FTP signal across the group. We applied a threshold of *P* value < 9.9e−5 to each correlation matrix, which corresponds to a family-wise error (FWE) corrected *P* value of 0.05 for each matrix. Lastly, in the PVC methods we used, medial and lateral orbitofrontal were combined into a single ROI, pars orbitalis, opercularis, and triangularis were combined into a single ROI, and rostral and caudal middle frontal were combined into a single ROI.

## Results

### Subject characteristics

A total of 134 ADNI participants met study criteria (Aβ positive, received tau-PET imaging, received MRI, and underwent the neuropsychology battery). Three subjects were excluded as their structural MRI did not successfully process through FreeSurfer or their MRI had substantial motion artifact, leaving us with 131 participants to examine. Of these participants, 55 had a diagnosis of cognitively normal, 46 with mild cognitive impairment, and 30 with AD. The average participant age was 77.8 (±7.3) years and 65 of the participants were female. Participant demographics and a summary of cognitive test scores are shown in Table 1.

**Table 1:** Subject demographics and cognitive test scores by diagnostic group

Variable	CN	MCI	AD	All subjects	Domains tested
*N* (%female)	55 (56.3%)	46 (39.1%)	30 (53.3%)	131 (49.6%)	
Age	77.3 (6.3)	77.5 (7.6)	79.0 (8.5)	77.8 (7.3)	
RAVLT learning	45.7 (10.9)	31.9 (9.0)	25.6 (9.9)	36.3 (13.1)	Learning
RAVLT delayed recall	7.7 (4.7)	2.8 (3.5)	1.5 (3.0)	4.6 (4.8)	Memory
LM immediate recall	15.0 (3.2)	9.8 (4.9)	6.0 (5.25)	11.1 (5.7)	Memory
LM delayed recall	14.0 (3.7)	7.5 (5.2)	2.8 (4.6)	9.1 (6.3)	Memory
Category fluency—animals	22.0 (5.1)	16.5 (5.2)	12.5 (4.5)	17.9 (6.3)	Language
MINT*	30.2 (2.4)	28.6 (3.9)	26.4 (5.1)	28.9 (3.8)	Language
Clock drawing	4.8 (0.5)	4.2 (0.9)	3.8 (1.6)	4.4 (1.1)	Visuospatial
Clock copying	4.7 (0.8)	4.7 (0.5)	4.3 (1.1)	4.6 (0.8)	Visuospatial
Trail Making Test A^†^	31.9 (7.7)	41.3 (19.4)	60.3 (36.2)	41.7 (24.0)	Executive function and processing speed
Trail Making Test B^†^	81.6 (40.2)	128.5 (82.2)	202.2 (99.3)	125.7 (86.6)	Executive function and processing speed

Values are mean ± SD unless otherwise noted

*Abbreviations*: *N* number of participants, *SD* standard deviation, *RAVLT* Rey Auditory Verbal Learning Test, *LM* logical memory, *MINT* Multilingual Naming Test, *CN* cognitively normal, *MCI* Mild Cognitive Impairment, *AD* Alzheimer’s disease

*Since MINT was added to the ADNI neuropsychology battery only recently, MINT scores were missing for 26 of our study participants

^†^In the Trail Making Test, a higher score denotes worse performance

### Associations between tau and cognitive function

#### Learning and Memory

**Fig. 2:**
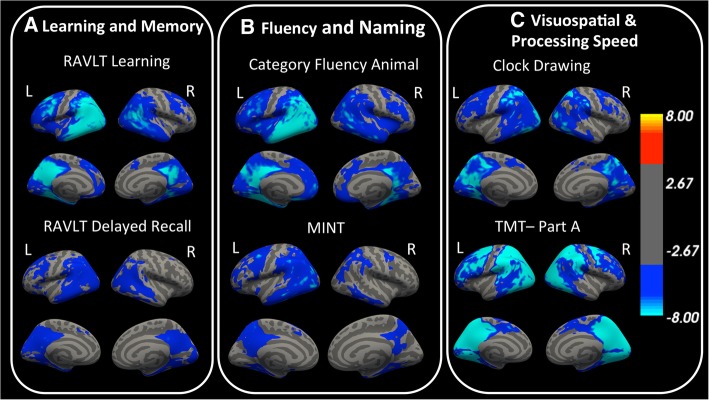
Surface-based analysis demonstrates that FTP retention is associated with cognitive scores. **a** Performance on learning and memory tests are associated with tau in temporal, parietal, and lateral frontal cortex. **b** Fluency and naming abilities are related to tau in temporal, parietal, frontal, and lateral occipital cortex, with left-sided laterality. **c** Visuospatial function and processing speed are also related to tau in lateral frontal, lateral temporal, and parietal cortex. Plots are in -log(*P* value) scale and vertex values range from -log (4) to -log (8). Cool colors indicate that higher FTP retention is related to worse cognitive performance. For multiple-comparisons correction, a cluster-forming threshold of -log(*P* value) > 4 or *P* value < 0.0001, and a cluster-wise threshold *P* value < 0.05 were used. Results from vertex-wise analyses with LM, Clock Copying, and TMT part B displayed similar associations as RAVLT, Clock Drawing and TMT part A, respectively, and can be found in Additional file 1: Figure S1a. Abbreviations: RAVLT, Rey Auditory Verbal Learning Test; MINT, Multilingual Naming Test; TMT, Trail Making Test

In general, worse scores on the RAVLT and LM were associated with more FTP binding (Fig. 2a). Visual inspection of the statistical maps revealed that lower cognitive scores were associated with FTP in medial, inferior, lateral temporal cortex and also in medial and lateral parietal cortex. Notably, there were stronger associations in frontal regions for RAVLT learning and LM immediate recall than for RAVLT delayed recall and LM delayed recall, respectively.

#### Fluency and naming

Lower performance on category fluency and MINT was also associated with greater FTP, as shown in Fig. 2b. Significant correlations were present in the left frontal cortex and temporal, parietal, and lateral occipital cortex and were mostly left lateralized (Additional file 1: Figure S3).

#### Visuospatial, executive function, and processing speed

Compared to learning, memory, fluency, and naming tests, the clock copying and TMT performance had greater associations with FTP in posterior regions, including more superior parietal cortex (Fig. 2c). In addition, clock drawing, clock copying, and TMT part A also involved the medial temporal cortex to a lesser extent (Fig. 2c) than tests of learning, memory, and language. Compared to TMT part A, the association between FTP and TMT part B had more inferior and medial temporal lobe involvement.

### Associations between thickness and cognitive function

#### Learning and memory

**Fig. 3:**
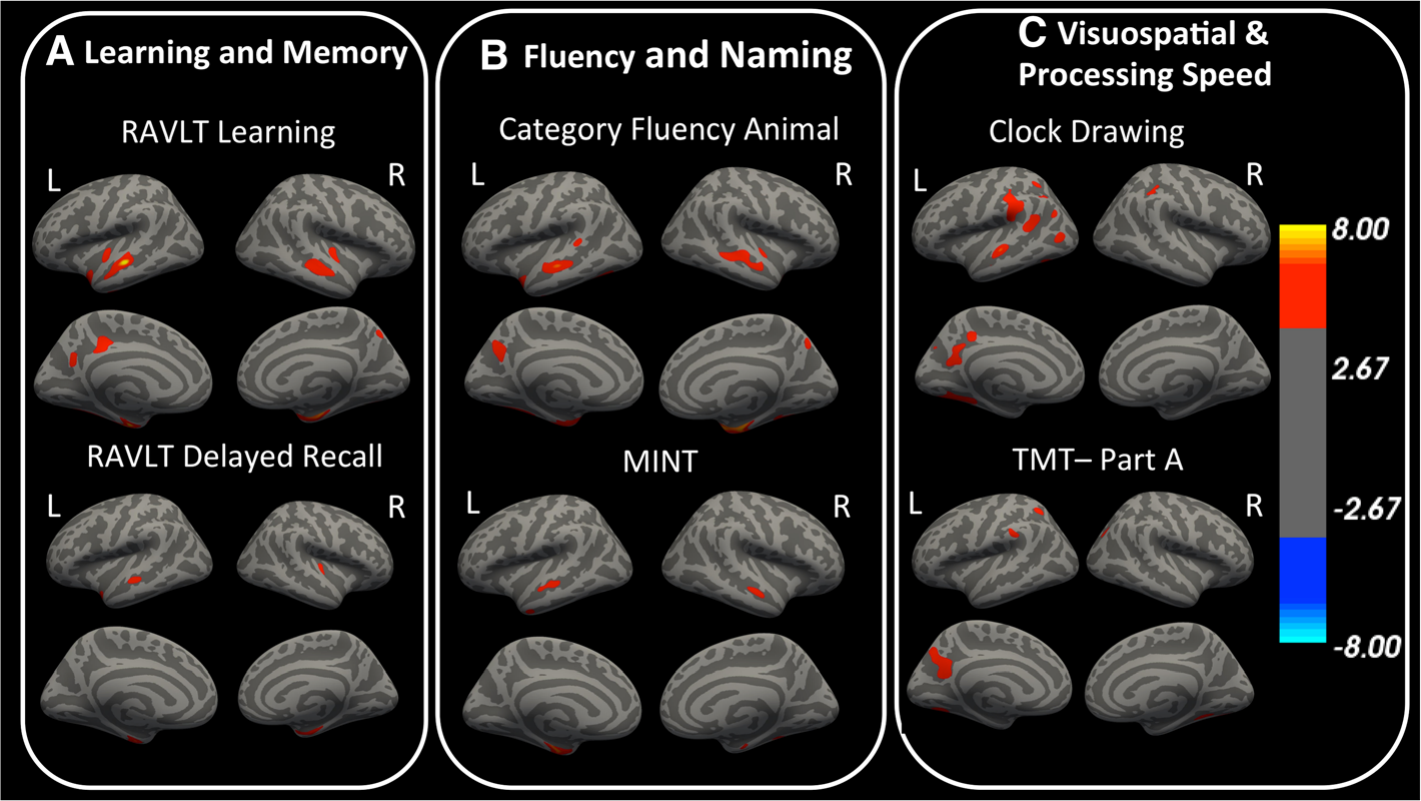
Surface-based analysis demonstrates that cortical thickness is associated with cognitive scores. **a** Learning and memory abilities are related to cortical thickness in temporal cortex and medial parietal cortex. **b** Performance on fluency and naming tests are associated with thickness in temporal and medial parietal cortex. **c** Visuospatial function and processing speed are related to thickness in inferior and lateral temporal, and medial and lateral parietal cortex. Plots are in -log(*P* value) scale and vertex values range from -log (4) to -log (8). Warm colors indicate that thicker cortex is related to better cognitive performance. For multiple-comparisons correction, a cluster-forming threshold of -log(*P* value) > 4 or *P* value < 0.0001, and a cluster-wise threshold *P* value < 0.05 were used. Results are shown for the same cognitive tests as Fig. 1. Results for the rest of the cognitive tests can be found in Additional file 1: Figure S1b. Abbreviations: RAVLT, Rey Auditory Verbal Learning Test; MINT, Multilingual Naming Test; TMT, Trail Making Test

Worse scores on the RAVLT and LM tests were associated with thinner medial and lateral temporal cortices and with medial parietal cortices (Fig. 3a).

#### Fluency and naming

The topographies of the relationships between thickness and category fluency, and between thickness and MINT, were similar to the topographies from the thickness-RAVLT and thickness-LM associations (Fig. 3b). The thickness-category fluency association involved more medial parietal cortex than the thickness-MINT association.

#### Visuospatial, executive function, and processing speed

Poorer clock drawing and TMT scores were associated with reduced thickness in medial and parietal cortex, and inferior temporal cortex (Fig. 3c). Clock copying was not significantly related to thickness anywhere in the cortical mantle.

Visual assessment of the statistical maps from Figs. 1 and 2 revealed that the association between cortical thickness and cognition, are less widespread than those between FTP and cognition. Finally, in the diagnosis-stratified surface-based analyses, we found no robust associations between tau and cognition or between thickness and cognition.

### Domain-specificity analysis

**Fig. 4:**
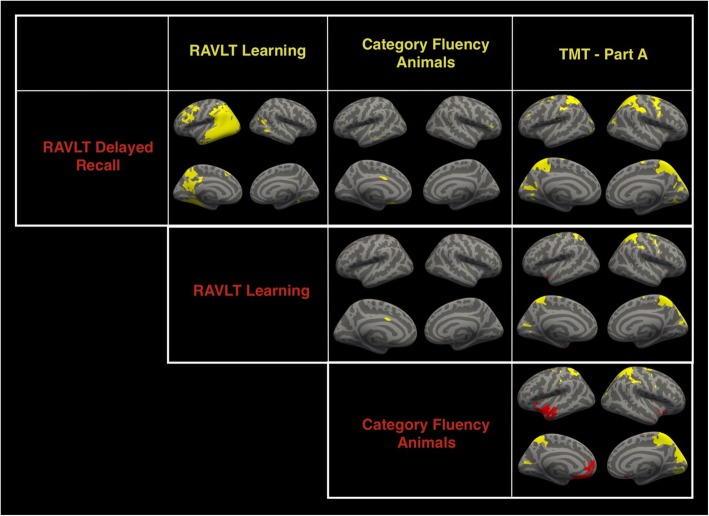
Meng’s test analyses show that FTP exhibits domain-specific relations with cognition. Yellow indicates that the cognitive measure noted in the column had a stronger association with FTP at that vertex than the cognitive measure noted in row (*P* value < .005). Red indicates that the cognitive measures noted in the row had a stronger association with FTP at that vertex than the cognitive measures noted in the column (*P* value < 0.005). Meng’s test was limited to regions of the cortex that were significant after cluster-wise correction in the FTP-cognition or thickness-cognition analysis. Abbreviations: RAVLT, Rey Auditory Verbal Learning Test; TMT, Trail Making Test

Findings from Meng’s test domain specificity analyses are shown in Fig. 4. Pairwise comparisons between tau-cognitive function associations revealed that the association between FTP and TMT part A was stronger in the superior parietal areas of the cortex than the other FTP-cognition associations. The relationship between FTP and RAVLT learning was stronger than that between FTP and RAVLT delayed recall in the left lateral temporoparietal cortex, left medial parietal cortex, left posterior cingulate cortex, left inferior temporal cortex, and the left dorsolateral frontal cortex. The FTP-category fluency association was stronger in the left anterior temporal lobe, bilateral medial orbitofrontal, and bilateral insula than the FTP-TMT part A association.

The results from the domain-specificity analyses from the thickness-cognition associations are displayed in Additional file 1: Figure S2. In brief, Meng’s test analyses did not elucidate any notable domain-specific correlation patterns between thickness and cognitive function.

### Region-of-interest analysis

**Fig. 5:**
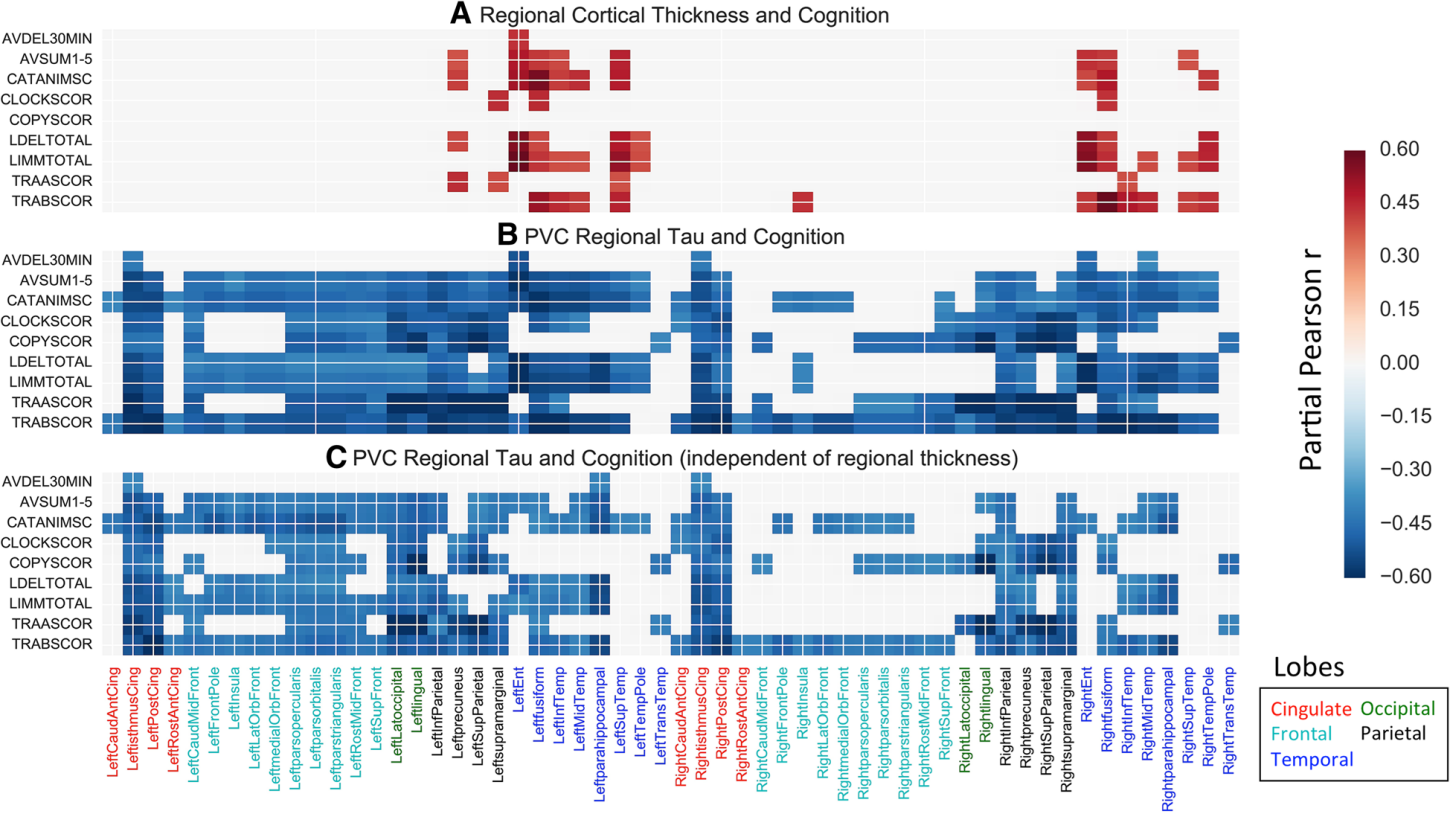
Region-of-interest analyses showing the relationship between regional tau and cognition, and between regional thickness and cognition. The value in each cell of the matrix shows the partial correlation between **a** regional thickness and cognition, **b** regional PVC-FTP and cognition, and **b** between regional PVC-FTP and cognition, after accounting for regional thickness. The association of cognition with regional PVC-FTP or regional thickness recapitulate our vertex-wise findings. Matrices are displayed at a threshold of *P* value < 9.9e−5. In **b**, the columns for medial orbitofrontal and lateral orbitofrontal are identical, the columns for pars orbitalis, opercularis, and triangularis are identical, and the columns for rostral and caudal middle frontal are identical. This is a consequence of the PVC methods we used (see the “Methods” section) which combine individual FreeSurfer ROIs into larger ROIs. Abbreviations: PVC, Partial Volume Corrected; ROI, region-of-interest; AVDEL30MIN, RAVLT Delay; AVSUM15, RAVLT Learning; CATANIMSC, Category Fluency—Animals; CLOCKSCOR, Clock Drawing; COPYSCOR, Clock Copying; LDELTOTAL, LM Delay; LIMMTOTAL, LM Immediate; TRAASCOR, TMT Part A; TRABSCOR, TMT Part B

As was the case for the vertex-wise analyses, the ROI analyses showed that FTP was related to performance on each cognitive test in broad areas of the cortex (Fig. 5b). Furthermore, the ROI FTP-cognition associations showed similar topographies as the vertex-wise FTP-cognition associations. Temporal, parietal, and posterior portions of the cingulate were consistently involved across tests. However, TMT, clock drawing, and clock copying tau showed a relatively greater predilection for posterior parts of the cortex compared to tests of learning, memory, fluency, and naming tests. Regional cortical thickness was also related to cognition, though the associations were much more sparse than the ROI FTP-cognition relations (Fig. 5a). The regional thickness associations were limited to temporal cortex and left parietal cortex.

Lastly, even after accounting for regional thickness, ROI FTP remained significantly associated with cognition (Fig. 5c), though the magnitudes of the partial correlations were reduced.

## Discussion

We used flortaucipir-PET and structural MRI to assess the relationships of tau and cortical thickness with performance in several domains of cognition. We found that worse cognition is related to greater tau burden and thinner cortex. We also found that the tau-cognition relationships were present in a domain-specific manner and were more widespread than the thickness-cognition relationships. We discuss our findings in the context of previous studies that have examined in vivo tau, atrophy, and cognition.

The implication of medial temporal, medial parietal, and lateral parietal tau load with memory and with global cognition has been well documented and replicated by several groups since the development of in vivo tau methods [13, 47]. Our findings are congruent with those previous studies and build upon them in a number of ways. For example, in our surface-based tau analyses, we found relatively greater left prefrontal cortex involvement in the RAVLT learning than the RAVLT delayed recall and also greater left prefrontal involvement in the LM immediate recall than LM delayed recall. This first highlights that the association between tau and AD-related learning and memory impairment is not restricted to temporal and parietal cortex. Furthermore, the implication of left prefrontal cortex is consistent with data from functional imaging and lesion studies that have localized verbal learning and verbal working memory to left frontal cortex [48, 49], suggesting that the specific location of tau deposition relates to specific aspects of cognitive loss.

The regional specificity between tau and cognitive impairment has been demonstrated in the context of early-onset, non-amnestic forms of AD, where clinical variants exhibit different topographies of tau that are consistent with the clinical phenotype [24, 25]. There is now also mounting evidence that, even in late-onset, amnestic AD, tau burden regionally associates with performance on domain-specific tests of cognitive function above and beyond learning and memory [27] and that this association maps onto the cortical circuitry underlying the respective cognitive domain. This notion is supported by the significant associations between tau and tests of language, processing speed, visuospatial and executive functions, and semantic knowledge that we found in our study. Although there was spatial overlap between cognitive tests in their associations with tau, particularly in early Braak regions, there was also considerable heterogeneity. This includes left-hemispheric laterality for tests of language and stronger superior parietal involvement in tests of processing speed and constructional ability compared to tests of memory and language. These patterns of tau-cognition associations mapped closely with the cortical systems that are believed (based on functional imaging and lesion studies) to support the specific cognitive functions tested. The finding that TMT parts A and B displayed stronger associations in parietal cortex compared to tests of memory and language, for example, is congruent with fMRI studies that have shown that tasks involving numbers rely on parietal cortex [50] and that the set-shifting necessary for successful completion of TMT part B is supported by activation of intraparietal sulcus [51]. Taken together, our findings from surface-based analyses with tau-PET are aligned with recent studies of patients with early-onset [24, 25] [52] and late-onset AD [27] that have demonstrated that performance in various cognitive domains associates with tau along distinct topographic patterns.

Cortical thickness also correlated with performance across tests, and these associations were topographically similar to the tau-cognition associations. Both correlations involved temporal and parietal cortex across cognitive domains, whereas tests of processing speed and constructional abilities showed stronger correlations with tau in more posterior cortex than tests of memory and language. However, the thickness-cognition relationships were more spatially restricted than the tau-cognition relationships. Although the order in which disease-related tau and atrophy appear during aging and AD has been under discussion [53], models of AD pathophysiology have historically postulated primacy of tau pathology [54]. Our finding that the tau-cognition associations were more widespread than the atrophy cognition associations is consistent with this model, but future studies that assess parallel longitudinal atrophy and tau will help to clarify the temporal staging of these AD biomarkers.

Our results from the surface-based, vertex-wise analyses shown in Figs. 2 and 3 demonstrate that there are areas of the cortex where cognition is related to tau, but not to cortical thickness. This is recapitulated in our ROI analyses. We further showed in the ROI analyses that, even after taking regional thickness into account, PVC-FTP still predicted cognition. We interpret these findings to mean that tau may exert an influence on cognition via mechanisms that are independent of structural atrophy. There are several potential pathways through which tau may induce cognitive loss such as synaptic dysfunction and synaptic loss. Some of these alterations, which can now be estimated in vivo using PET [55], should be the focus of future studies to provide a better view of how the hallmark AD pathologies like tau drive decline and to realize new potential avenues for therapeutic intervention. We should note that the widespread topography of tau-cognition associations relative to thickness-cognition associations may be a consequence of deriving the tau and cortical thickness measures from different modalities. However, we applied a differential smoothing procedure to account for this and, moreover, a recent study by Bejanin and colleagues [25] also reported data suggestive of an atrophy-independent effect of tau on cognition, lending further credence to our interpretation.

Our findings have several practical implications. For example, a critical aim for designing a well-powered clinical trial is enrolling individuals that are on the verge of AD-related cognitive decline. Although our data are cross-sectional, the robust relation between tau and cognition may indicate that tau burden is a better and more sensitive predictor of imminent decline than is cortical thickness. There are several potential reasons as to why tau-PET may be a more sensitive tool for detecting decline. As we discussed above, tau may precede neurodegeneration [56] and begin to exert its affect on cognition prior to discernable atrophy. Furthermore, cross-sectional MRI-based estimates of atrophy may be affected by premorbid, inter-individual variability in thickness. Related to this, there is some evidence that atrophy measures derived from longitudinal MRI may offer predictive power beyond cross-sectional measures of atrophy in forecasting cognitive decline [57]. Thus, it will be important to incorporate longitudinal MRI if seeking to determine which imaging measures are most useful in clinical trial contexts. Lastly, because we focused on cortical patterns of tau and atrophy as they relate to cognition, we excluded hippocampal atrophy from our analyses. Hippocampal atrophy has been shown to be an indicator of imminent cognitive decline [58, 59] so estimates of hippocampal volume loss, in addition to cortical thinning, should be included in studies seeking to address the relative utilities of MRI and PET in predicting decline for powering clinical trials.

With further regard to clinical trials, there are now several compounds in the AD pipeline whose mechanism of action involves targeting tau pathology [60]. For these trials, it will be critical to identify individuals that are currently undergoing, or are expected to undergo, cognitive changes that are related to tau, rather than changes due to non-AD neurodegenerative, neuropsychiatric, or normal aging processes. Our data first demonstrate that noninvasive and economical neuropsychological tests like those used in ADNI can detect tau-related changes, supporting their potential utility as a screening tool. In addition, given that tau correlated with cognition in several domains, our data highlight the importance of designing neuropsychological batteries or composites for clinical trials that evaluate functions beyond memory.

Some limitations to our study should be acknowledged. FTP has been shown to exhibit off-target binding to choroid plexus in the areas adjacent to the hippocampus [61], to monoamine oxidases [62], and others. Newer generations of radiotracers that bind more specifically to tau [63] will minimize this problem in future studies. Another limitation is that our sample of participants is relatively skewed to early disease stages. This may have produced a limited range in cognitive measures and FTP signal, and hence, could have limited our ability to detect some tau-cognition and thickness-cognition relationships that may be present in AD. We were further limited by the brief battery in ADNI. Future studies with more extensive testing, for example, testing memory with nonverbal as well as verbal stimuli, will allow for broader understanding of tau-cognition relationships. The final shortcoming of our study is the small sample size, which potentially explains the failure to find meaningful tau-cognition and thickness-cognition associations in the diagnosis-stratified analyses. Future studies in larger cohorts may demonstrate relationships within diagnostic groups, helping to explain the staging of tau-atrophy relationships across the spectrum of the disease.

## Conclusions

In a group of individuals along the AD spectrum, we found that cognitive decline in specific domains parallels the deposition of tau into the cortical systems that are thought to be responsible for subserving those domains. Our vertex and ROI analyses also revealed that there are areas of cortex where cognition is related to tau, but not cortical thickness, suggesting that tau may have an effect on cognition via mechanisms that are, at least in part, independent of significant atrophy. These data are consistent with previous studies and offer further insights into the pathophysiology underlying cognitive impairment in AD.

## Additional file

Additional file 1: Method S1. Assessing laterality of FTP-cognition and thickness-cognition associations. Method S2. Examining the affect of Aβ on FTP-cognition and thickness cognition associations. **Figure S1.** Results from vertex-wise associations between FTP and cognitive scores, and between cortical thickness and cognitive scores. **Figure S2.** Results from Meng’s test analyses on cortical thickness-cognition correlation maps. **Figure S3.** Distribution of Laterality Indices (LI) from each FTP-cognition and thickness-cognition vertex-wise analysis. **Figure S4.** Regional PVC-FTP and regional thickness are associated with cognition independent of Aβ (DOCX 115582 kb)

## Data Availability

All neuroimaging and neuropsychology data that were used in this investigation is available online at the ADNI data repository (adni.loni.usc.edu).

## Abbreviations

AD: Alzheimer’s disease
ADNI: Alzheimer’s Disease Neuroimaging Initiative
Aβ: Amyloid-β
CN: Cognitively Normal
FDG: Fluorodeoxyglucose
FTP: Flortaucipir
LM: Logical Memory
MCI: Mild Cognitive Impairment
MINT: Multilingual Naming Test
MRI: Magnetic resonance imaging
PET: Positron emission tomography
PVC: Partial volume correction/corrected
RAVLT: Rey Auditory Verbal Learning Test
ROI: Region of interest
SD: Standard deviation
SE: Standard error
SUVr: Standard uptake value ratio
TMT: Trail Making Test
