# Supplementary material for: Tau and atrophy: domain-specific relationships with cognition
Source: Alzheimers Res Ther. 2019 Jul 27;11:65. doi: 10.1186/s13195-019-0518-8 (PMC6661099; doi:10.1186/s13195-019-0518-8)
Supplement: Supplementary file 1 — Method S1. Assessing laterality of FTP-cognition and thickness-cognition associations. Method S2. Examining the affect of Aβ on FTP-cognition and thickness cognition associations. Figure S1. Results from vertex-wise associations between FTP and cognitive scores, and between cortical thickness and cognitive scores. Figure S2. Results from Meng’s test analyses on cortical thickness-cognition correlation maps. Figure S3. Distribution of Laterality Indices (LI) from each FTP-cognition and thickness-cognition vertex-wise analysis. Figure S4. Regional PVC-FTP and regional thickness are associated with cognition independent of Aβ (DOCX 115582 kb) [file 13195_2019_518_MOESM1_ESM.docx]

**Tau and atrophy: domain-specific relationships with cognition**

**Additional file 1**

Leonardino A. Digma BA, John R. Madsen BS, Emilie T. Reas PhD, Anders M. Dale PhD, James B. Brewer MD, PhD, Sarah J. Banks PhD

**Method S1.** Assessing laterality of FTP-cognition and thickness-cognition associations.

**Method S2.** Examining the affect of Aβ on FTP-cognition and thickness cognition associations.

**Figure S1.** Results from vertex-wise associations between FTP and cognitive scores, and between cortical thickness and cognitive scores

**Figure S2.** Results from Meng’s test analyses on cortical thickness-cognition correlation maps.

**Figure S3.** Distribution of Laterality Indices (LI) from each FTP-cognition and thickness-cognition vertex-wise analysis.

**Figure S4.** Regional PVC-FTP and regional thickness are associated with cognition independent of Aβ

**References**

**Method S1. Assessing laterality of FTP-cognition and thickness-cognition associations.**

Visual inspection of the statistical maps from the surface-based analysis revealed that some of FTP-cognition associations demonstrated stronger associations in the left hemisphere than the right (Figure 1 and Supplementary Figure 2). We further assessed the laterality in FTP-cognition and thickness-cognition associations by calculating laterality indices (LI). The LI was computed as follows [1]:

$$LI=f\times\frac{Q_{LH}-Q_{RH}}{Q_{LH}+Q_{RH}}$$

where f is an arbitrary constant that places minimum and maximum bounds on LI, set at f=100 here. Q_LH_ and Q_RH_ are measures for the left and right hemisphere, respectively. In our study, we determined Q in each hemisphere by calculating the hemisphere’s median z-statistic from the statistical map of each surface-based analysis (i.e. the statistical maps displayed in Figure 1, Figure 2, and Supplementary Figure 2). The distribution of LIs are reported in Supplemental Figure 3.

**Method S2.** Examining the affect of Aβ on FTP-cognition and thickness cognition associations.

We were interested in whether to relationships between FTP and cognition, and between thickness and cognition were related to global amyloid-β (Aβ) burden. To probe this question, we performed the following ROI analysis. We first computed partial correlations between regional PVC-FTP and cognitive test score, after regressing each variable onto age, sex, education, and time-delay between scan and cognitive test. Then, we again computed partial correlations between regional PVC-FTP and cognitive test score, after regressing each variable onto global Aβ, age, sex, education, and time-delay between scan and cognitive test. This analysis was repeated for regional thickness instead of regional PVC-FTP. Results from our ROI analyses are displayed in correlation matrices in Supplementary Figure 4 and a threshold of *P* value<9.9e-5 was applied to the matrices, which corresponds to a family-wise error corrected *P* value of 0.05 for each matrix.

The participants in our study received two different tracers to assess extent of Aβ pathology (a majority received florbetapir and the others received florbetaben) so we conducted this analysis in the subset (N=123) that received florbetapir. Global Aβ burden was calculated by the Jagust Lab at UC Berkeley using previously described methods [2] and these values are publicly available on the ADNI data portal.

**Figure S1. Results from vertex-wise associations between A) FTP and cognitive scores, and between B) cortical thickness and cognitive scores.** Plots are in -log(*P* value) scale with cool colors indicating a negative association between FTP/thickness and cognitive score and warm colors indicating a positive association. Results are shown for the set of tests that are not included in Figure 1 of the main article. Clock copying was not related to cortical thickness anywhere in the cortical mantle.

**Figure S2. Results from Meng’s test analyses on cortical thickness-cognition correlation maps.** Yellow indicates that the cognitive measure noted in the column had a stronger association with thickness at that vertex than the cognitive measure noted in row (vertex-wise, *P* value<.005). Red indicates that the cognitive measures noted in the row had a stronger association with thickness at that vertex than the cognitive measures noted in the column (vertex-wise, *P* value<.005). Meng’s test was limited to regions of the cortex that were significant after cluster-wise correction.

**Figure S3. Distribution of Laterality Indices (LI) from each FTP-cognition and thickness-cognition vertex-wise analysis.** In the FTP-cognition associations, tests that involved a greater language component tended to have stronger tau-cognition associations in the left hemisphere than the right. No discernable pattern was observable from the distribution of LI indices from the statistical maps of thickness-cognition associations. Abbreviations: AVDEL30MIN, RAVLT Delay; AVSUM15, RAVLT Learning; CATANIMSC, Category Fluency - Animals; CLOCKSCOR, Clock Drawing; COPYSCOR, Clock Copying; LDELTOTAL, LM Delay; LIMMTOTAL, LM Immediate; MINTTOTAL, Multilingual Naming Test; TRAASCOR, TMT Part A; TRABSCOR, TMT Part B.

**Figure S4. Regional PVC-FTP and regional thickness are associated with cognition independent of Aβ**. The value in each cell of the matrix shows the partial correlation (A) between regional thickness and cognition, (B) between regional thickness and cognition after accounting for global Aβ, (C) between regional PVC-FTP and cognition, and (C) between regional PVC-FTP and cognition, after accounting for global Aβ. Panels A and C are recreations of Figure 5A and 5b, respectively, from the main text and are shown here to demonstrate that global Aβ has little influence on the association of cognition with regional thickness and PVC-FTP. Abbreviations: PVC, Partial Volume Corrected; ROI, region-of-interest: AVDEL30MIN, RAVLT Delay; AVSUM15, RAVLT Learning; CATANIMSC, Category Fluency - Animals; CLOCKSCOR, Clock Drawing; COPYSCOR, Clock Copying; LDELTOTAL, LM Delay; LIMMTOTAL, LM Immediate; MINTTOTAL, Multilingual Naming Test; TRAASCOR, TMT Part A; TRABSCOR, TMT Part B.

**References**

[1]	Rabinovici GD, Jagust WJ, Furst AJ, Ogar JM, Racine CA, Mormino EC, et al. Abeta amyloid and glucose metabolism in three variants of primary progressive aphasia. Ann Neurol 2008;64:388–401. doi:10.1002/ana.21451.

[2]	Landau SM, Breault C, Joshi AD, Pontecorvo M, Mathis CA, Jagust WJ, et al. Amyloid-β imaging with Pittsburgh compound B and florbetapir: comparing radiotracers and quantification methods. J Nucl Med Off Publ Soc Nucl Med 2013;54:70–7. doi:10.2967/jnumed.112.109009.
